# Persulfidation alters gene regulatory programs and promotes endothelial specification

**DOI:** 10.1016/j.redox.2025.103926

**Published:** 2025-11-28

**Authors:** Janina Wittig, Ran Xu, Fredy Delgado Lagos, Maria-Kyriaki Drekolia, Boran Zhang, Ioannis Theodorou, Yunyun Chen, Yali Du, Lavanya Gupta, Cui Hanyu, Li Yuanyuan, Cheng Bo, Stefan Günther, Ilka Wittig, Roxana Ola, Jiong Hu, Sofia-Iris Bibli

**Affiliations:** a European Center for Angioscience, Department of Vascular Dysfunction, Medical Faculty Mannheim, Heidelberg University, Mannheim, Germany; b Goethe University, Institute for Vascular Signalling, Centre for Molecular Medicine, Frankfurt am Main, Germany; c Department of Histology and Embryology, School of Basic Medicine, Tongji Medical College, Huazhong University of Science and Technology, Wuhan, China; d German Center of Cardiovascular Research (DZHK), Germany; e Deep Sequencing Platform, Max Planck Institute for Heart and Lung Research, Bad Nauheim, Germany; f Goethe University, Functional Proteomics, Institute for Cardiovascular Physiology, Centre for Physiology, Frankfurt am Main, Germany; g European Center for Angioscience, Experimental Pharmacology Mannheim, Medical Faculty Mannheim, Heidelberg University, Mannheim, Germany

**Keywords:** Sulfide supplementation, Endothelial commitment, Stem cells, Persulfidation

## Abstract

Endogenously generated sulfides are conserved among species and tissues and exert multiple effects through diverse mechanisms. Although sulfides have been linked to cell fates, their role in pluripotent stem cell commitment remains unknown. Here we discovered that during directed differentiation of induced pluripotent stem cells, endogenous sulfide levels drop in all three germ layers, with the mesodermal lineage exhibiting the lowest capacity to generate these species at early specification events. Addition of a rapid releasing sulfide donor in iPSCs or mesodermal cells did not affect the redox surveillance mechanisms, however, it altered persulfidation and transcription of cell fate commitment pathways. In particular, sulfide supplementation in pluripotent stem cells reduced cell differentiation processes by preserving the activity of the stem cell transcription factors OCT4. In contrast, supplementation of sulfide during mesodermal lineage specification promoted persulfidation and activated the WNT signaling as well as enriched the activity of the ETS transcription factor family, resulting in increased transcription of angiogenic and vessel morphogenesis genes. Sulfide addition during the development of vascular organoids enhanced blood vessel morphogenesis. Taken together, these data position protein persulfidation as a timing-dependent regulator that preserves pluripotency prior to commitment but subsequently biases mesoderm toward endothelial specification, thereby emerging as a tractable redox modification for engineering stem cell fate and vascularization.

## Highlights

• Mesodermal commitment is characterized by reduced endogenous sulfide levels.
• Persulfidation shifts across states: it marks pluripotency factors in iPSCs, then targets WNT/β-catenin after mesoderm entry.
• Sulfide supplementation in iPSCs preserves pluripotency, boosting OCT4 activity.
• Sulfide boosts endothelial specification in mesodermal cells by activating WNT signaling and enhancing ETS factor activity.

## Graphical abstract

**Figure:**
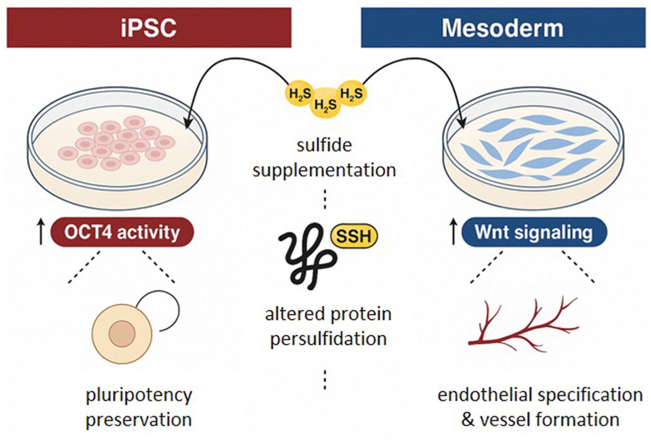

## 1 Introduction

Endogenous sulfide biosynthesis plays important roles in maintaining organismal homeostasis, while loss of sulfide metabolism has been linked to several human diseases [1]. Intracellular sulfide levels regulate protein persulfidation, which is a highly conserved post-translational protein modification of the redox-sensitive amino acid cysteine [2]. Temporal dynamics of protein persulfidation during aging have been proposed and recent studies highlight the potential of such conserved regulatory mechanisms against protein overoxidation and degradation [2,3]. While the role of persulfidation has been extensively described in terminally differentiated cells and disease conditions [4,5], its role in cell fate commitment remains unknown. Only a few studies have proposed a significant role of endogenous sulfides in the regulation of stem cell phenotypes. In particular, sulfide deficiency halts bone marrow mesenchymal stem cell differentiation [6] and survival [7] through aberrant intracellular Ca^2+^ influx, which results in osteoporotic phenotypes. In contrast, sulfide supplementation enhances the effectiveness of mesenchymal stem cell therapy in heart failure models [8] and promotes the neuronal differentiation [9]. Yet, the direct role of sulfide supplementation in pluripotent stem cell commitment and the potential interplay between protein persulfidation and gene regulation remains elusive. Pluripotent stem cells possess the remarkable ability to self-renew and generate all cell types present in both the embryo and adult organisms. Molecular aberrations in the pluripotency state can lead to catastrophic consequences resulting in the termination of embryogenesis [10], while loss of tissue‐resident stem cells in adulthood impacts on organ homeostasis and repair after injury [11]. As such, here we aimed to identify the impact of protein persulfidation in pluripotent stem cell commitment and cell specification. Our data suggest that particularly mesodermal commitment is characterized by a reduced sulfide flux. Sulfide supplementation in pluripotent stem cells as well as in mesodermal specified cells results in persulfidome perturbations which alter transcriptional programs. Altered persulfidome landscape in the mesodermal lineage enhances endothelial cell specification and promotes vessel morphogenesis.

## 2 Materials and methods

### 2.1 iPSCs culture, differentiation and treatment

All experiments were performed in the human induced pluripotent stem cell (iPSC) lines WTSIi072-A (Sex: male, EBiSC), WTSIi081-A (Sex: male, EBiSC) and hiPS-G1 (Sex: female, kindly donated from the Institute for Pharmacology and Toxicology at the University Medical Center in Göttingen). All iPSC lines were Sendai virus-induced and originated from healthy human fibroblasts. All iPSCs were cultured under feeder-free conditions on 0.8 % matrigel-coated plates (Corning) in TeSR-E8 medium (STEMCELL Technologies) at 37 °C and 5 % CO_2_ and were tested for mycoplasma contamination on a weekly basis. iPSCs were passaged by incubating with Versene solution (Gibco) for 5 min at room temperature, resuspending in TeSR-E8 medium containing 5 μmol/L ROCK inhibitor Y-27632 (Stemgent) and seeding in new 0.8 % matrigel-coated plates.

For mesodermal induction, iPSCs were grown on matrigel-coated plates until 80 % confluency and then treated for 48 h with mesodermal induction medium (RPMI 1640 medium with GlutaMAX, 1 mmol/L sodium pyruvate, 2 % B27 supplement (all Gibco), 200 μmol/L l-ascorbic acid 2-phosphate sesquimagnesium salt hydrate (Sigma-Aldrich)) containing 6 μmol/L CHIR99021 (STEMCELL Technologies), followed by 48 h in mesodermal induction medium containing 3 μmol/L CHIR99021 [12].

Cells in the iPSC condition were treated for 48 h with 1, 5 or 10 μmol/L of the H_2_S donor Na_2_S_3_ (Dojindo). To examine the effect of H_2_S supplementation on mesodermal lineage commitment, the same concentrations of Na_2_S_3_ were added after 48 h of mesodermal induction for further 48 h. Fresh donor was added every 24 h.

### 2.2 ^34^S polysulfide measurements

Polysulfides were detected following labelling of the respective studied groups with a^34^S–^13^C-cystine (#CSLM-11349-PK, Cambridge 451 Isotope Laboratories, custom synthetized) using ultra-performance liquid chromatography-mass spectroscopy as previously described [13].

### 2.3 Persulfidation proteomics

The qPerS-SID assay for the detection of persulfidated sites was used as previously described [14].

### 2.4 Persulfidation array and immunostaining

For immunofluorescent labeling of persulfidation, cells were cultured and treated in tissue culture μ-8 well-slides (ibiTreat, Ibidi, Martinsried, Germany). Samples were fixed with 4 % ROTI Histofix (Carl Roth) for 15 min at room temperature (RT) and subsequently incubated with 1 mmol/L NBF–Cl in PBS for 90 min at 37 °C. Cells were washed five times with 0.5 % Triton X-100 in PBS, of which the last washing step was over night at 4 °C with agitation. Samples were incubated with Daz2:Cy5 click mix (1 mmol/L Daz2 (Cayman Chemical), 100 μmol/L Cyanine5 alkyne (Lumiprobe), 0.2 mmol/L copper(II)-TBTA complex (Lumiprobe) and 4 mmol/L ascorbic acid made in situ, were added sequentially in PBS) for 1 h at 37 °C. For the negative control, cells were incubated with Daz2:Cy5 click mix prepared without Daz2. After seven washes with 0.5 % Triton X-100 in PBS, cells were incubated with Phalloidin Alexa Fluor 546 (Thermo Fisher Scientific, 1:1000) in 0.5 % Triton X-100 in PBS for 2–5 h at RT. Thereafter the cells were incubated with a DAPI solution (10 ng/mL DAPI in 0.5 % Triton X-100 in PBS) for 30 min at RT. After another three washing steps with 0.5 % Triton X-100 in PBS, the cells were mounted with fluorescent mounting medium (Dako, Glostrup, Denmark) and imaging was performed immediately using the Zeiss LSM 780 (Jena, Germany) confocal microscope with a 63× objective (Plan-Apochromat 63×/1.40 Oil DIC M27) and ZEN software (Zeiss, Jena, Germany). Non-sulfhydrated thiols were visible at 488 nm (NBF-adducts) and the sulfhydration signal was visible at 633 nm (Daz2:Cy5).

### 2.5 RNA sequencing and bioinformatics analysis

RNA was isolated using the RNeasy® Plus Universal Mini Kit (QIAGEN) according to the manufacturer's instructions. In brief, cells were lysed in Trizol with gDNA Eliminator and separated using chloroform. The RNA in the aqueous phase was precipitated using isopropanol and transferred to RNeasy® mini spin columns. The RNA was washed with RWT and RPE and eluted in RNase and DNase free water. RNA sequencing was performed and analyzed as previously described [15] and enrichment analysis as well as transcriptional factor activities were analyzed as previously described [16]. Module activity scores were computed from log_2_-transformed, scaled expression matrices by averaging z-scores of genes belonging to curated TGF-β- or WNT/β-catenin-related gene sets retrieved from MSigDB (Hallmark, Reactome, and GO:BP collections). For each lineage and treatment combination, Pearson's correlation coefficients between TGF-β and WNT/β-catenin module scores were calculated together with 95 % confidence intervals and p-values using Fisher's z-transformation. In addition, linear models of the form WNT ∼ TGF × Treatment were fitted per lineage to test for treatment-dependent differences in slope (interaction term). Visualizations were generated in R using ggplot2 (version 3.5 or later). Scatterplots display regression lines for each treatment, coloured in green (Sol) and orange (Na_2_S_3_).

### 2.6 FUCCI lentiviral generation

The two Fluorescent Ubiquitination-based Cell Cycle Indicator (FUCCI) constructs mcherry-hCdt1(30/120)pCSII-EF and mAG-hGeminin(1/110)/pCSII-EF were purchased from Riken BRC Center (#RDB15273 and #RDB15268, Ibaraki, Japan). The plasmids containing the lentivirus backbones pMD2.G and psPAX2 were purchased from Addgene (#12259 and #12260, Watertown, USA).

Lentiviruses were generated as previously described [17]. In brief, HEK293T cells were transfected with plasmids containing one of the two FUCCI constructs and the two lentivirus backbones. The supernatant of the cells containing the viral particles was collected and filtered through a 0.2 μm-filter. Medium containing viral particles was added to iPSC culture medium in a 1:2 ratio containing 8 μg/mL polybrene (#28728-55-4, Santa Cruz). The medium was changed 24 h after transduction to normal iPSC culture medium.

### 2.7 OCT4 reporter assay

Human OCT4 transcriptional activity was measured using the OCT4 CR4 pGreenFire Response Reporter assay (System Biosciences, Palo Alto, CA). Briefly, cells were transduced with the OCT4 CR4 pGreenFire lentiviral reporter, which places GFP and firefly luciferase under the control of OCT4 response elements. Following transduction and a 72 h recovery period and a subsequent treatment with 5 μmol/L Na_2_S_3_ for additional 24 h, reporter activity was quantified by GFP expression and by a luciferase assay using a standard luminometer. Reporter signal was normalized to total cell number to account for differences in cell viability and transduction efficiency.

### 2.8 Live cell cycle monitoring

Time lapse images were taken every 4 h using an IncuCyte with SX5 G/R Optical Module and the software version 2020B (Essen BioScience Inc.).

### 2.9 MitoSOX measurement

iPSCs and mesodermal cells were pre-treated for 48 h with and without Na_2_S_3_ (5 μmol/L) before the measurement of mitochondrial superoxide (MitoSOX) using the MitoSOX™ Red Mitochondrial Superoxide Indicator Kit (#M36008, Invitrogen). In brief, the cells were washed once with PBS and then stained with MitoSOX Red (1:5000) in PBS (+Ca^2+^ +Mg^2+^) for 30 min at 37 °C. Afterwards the cells were washed with PBS, collected using accutase, dissolved in 2 % BSA-PBS creating a single-cell suspension and immediately analyzed on a BD FACSCanto™ II (BD Biosciences). Live single cells were identified by FSC/SSC characteristics and single cells were identified using FSC-W/FSC-A. Data were analyzed using FlowJo V10 (TreeStar).

### 2.10 Immunoblotting and β-catenin stability

To assess β-catenin stability, iPSCs and mesodermal cells were cultured under control conditions (solvent) or with Na_2_S_3_ (5 μmol/L) for 24 or 48 h. Cells were collected and subjected to immunoblotting. Protein concentrations were determined using the Bradford assay. Equal amounts of proteins were solubilized in 3x SDS sample buffer (25.5 % glycerin, 6 % SDS, 188 mmol/L Tris/HCl pH 6.8, 60 mmol/L DTT, 0.006 % bromophenol blue), separated by SDS polyacrylamide gel electrophoresis (SDS-PAGE) using a Mini-PROTEAN Tetra Handcast System (Bio-Rad, Munich, Germany) and subjected to Western blotting using a Mini Trans-Blot Cell (Bio-Rad, Munich, Germany). Proteins were visualized by enhanced chemiluminescence using a commercially available kit (Amersham, Freiburg, Germany) and a Fusion Solo Western Blot Imaging instrument (Vilber, Eberhardzell, Germany). The following antibodies were used: mouse anti-3-nitrotyrosine (3-NT) mAb (1:1000, #ab110282, Abcam), mouse anti-vinculin mAb (1:1000, ThermoScientific, #MA5-11690), rabbit anti-pGSK3β (Ser9) (1:1000, Cell Signaling Technology, Cat. #9336, monoclonal, human reactive), anti–β-catenin (1:1000, Cell Signaling Technology, Cat. #8480, monoclonal, human reactive), rabbit anti-SMAD2/3 (1:1000, Cell Signaling Technology, Cat. #8685, monoclonal, human reactive) andrabbit anti p-SMAD2 (Ser465/467)/SMAD3 (Ser423/425) (1:1000, Cell Signaling Technology, Cat. #8828, monoclonal, human reactive). Relative protein levels were quantified by densitometry and normalized to vinculin.

### 2.11 FACS analysis

For flow cytometry, mesodermal cells treated with solvent or 5 μmol/L Na_2_S_3_ for 48 h were harvested and resuspended in PBS containing 0.1 % BSA. Live cells were identified using a viability dye (e.g. SYTOX™ Red, Thermo Fisher, Cat. #S34859) prior to fixation and permeabilization with BD Cytofix/Cytoperm buffer (BD Biosciences). Intracellular staining for mesodermal specification was performed using anti-EOMES antibody (Cell Signaling Technology, Cat. #14-4877-82, clone WD1928, human reactive). Data acquisition and cell sorting were carried out on a BD FACSAria II (BD Biosciences) and analysis was performed using FlowJo V10 (TreeStar).

### 2.12 Vascular organoid generation and treatment

Vascular organoids were generated as previously described [18]. During mesodermal induction in N2B27 medium containing CHIR99021 and BMP-4, the treatment of the aggregates with Na_2_S_3_ (5 μmol/L) started immediately with the addition of the medium and continued throughout the protocol until vascular organoids were formed.

### 2.13 Statistical analysis

Data are expressed as mean ± SEM and were analyzed for normal distribution using the Kolmogorov-Smirnov normality test. For normal distributed groups, statistical evaluation was performed using Student's t-test for paired data. One-way ANOVA (Tukey's multiple comparison tests) and two-way ANOVA (followed by Tukey's multiple comparisons test) were used where appropriate. Statistical tests and the number of biological replicates (n), as well as the number of technical replicates are described in the figure legend for each experiment. Values of p < 0.05 were considered as statistically significant.

## 3 Results

### 3.1 Pluripotency and lineage commitment display distinct endogenous sulfide levels and protein persulfidation

**Fig. 1:**
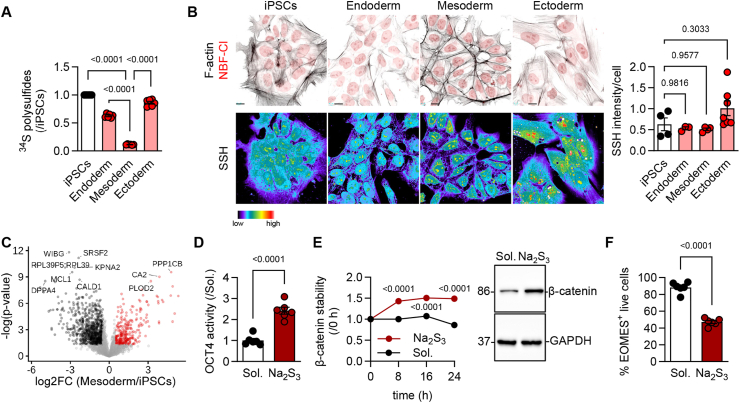
**Early mesodermal commitment is accompanied by reduced sulfide metabolic flux and altered protein persulfidation that maintains stemness.** **(A)**^34^S cystine derived polysulfides normalized to iPSCs. **(B)** False color images showing the intensity of the Daz2:Cy5 persulfidation signal iPSCs and in the three germ layers. bar = 10 μm. Fire pseudocoloring was used to distinguish the signal intensity. F-actin: black, NBF–Cl: red. **(C)** Volcano plot showing persulfidated proteins in iPSCs or cells directed to the mesodermal lineage. **(D)** OCT4 activity reporter assay in iPSCs treated with solvent (Sol.) or with 5 μmol/L Na_2_S_3_ for 24 h. **(E)** β-catenin stability in mesodermal cells treated with solvent (Sol.) or with 5 μmol/L Na_2_S_3_ for 24 h and representative immunoblot at 24 h. **(F)** Percentage (%) of EOMES-positive live cells in samples treated as in panel E. 3 iPSC lines were used in 2–3 technical replicates. Graphs include the average of all experiments per line. Paired Student's t-test (C, D, F), One-way ANOVA, Tukey's multiple comparison test (A, B), Two-way ANOVA, Tukey's multiple comparison test (E).

Induced pluripotent stem cells (iPSCs) exhibited higher ^34^S flux to sulfides compared to the lineage committed cells, with mesodermal lineage completely omitting sulfur donation (Fig. 1A). Subsequently, a modified dimedone-based immunofluorescence detection assay was used [2,14] to monitor the global cellular persulfidome as stem cells exited pluripotency towards the different germ layers. Notably, no significant global changes in protein persulfidation were detected among the tested groups (Fig. 1B). To identify protein-specific changes in persulfidation, next we used mass spectrometry to map persulfidated cysteines [14] in iPSCs maintained as stem cells and during early specification events, i.e. two days after directed differentiation towards the mesodermal lineage. This approach identified 1156 proteins with at least one persulfidated cysteine, with 533 of them being significantly different between the two groups (Fig. 1C–Table 1). Interestingly, persulfidated proteins enriched in the iPSCs included multiple direct inducers of stemness and regulators of the lineage commitment. In particular, our dataset identified that sulfides in iPSCs modify the primary regulators of pluripotency POU class 5 homeobox 1 (POU5F1) or octamer-binding transcription factor 4 (OCT4) [19] at cysteine 221 (localized within the DNA binding domain [20]) and developmental pluripotency associated 4 (DPPA4) [21], which are required for their self-renewal capacity. Such modification of POU5F1 was functional, as persulfidation in response to the rapid releasing polysulfide donor Na_2_S_3_ increased its activity, as identified by a CR4 pGreenFire Response Reporter assay [22] (Fig. 1D), which suggests that endogenous sulfides maintain stemness. Unexpectedly, while mesoderm directed cells lost the majority of the persulfidation targets, a shift to increased persulfidation of key stemness inhibitors and mesodermal lineage inducers was detected. In particular, persulfidation was significantly gained on cysteines of the bone morphogenic protein (BMP) and WNT/β-catenin signaling cascades. The most interesting targets include the glycogen synthase kinase 3β (GSK3β) (a negative regulator of the WNT/β-catenin signaling, which, when active, degrades β-catenin and inhibits mesodermal induction) at cysteine 14 and CTNNB1 (i.e. β-catenin itself which is indispensable for the formation of the primitive streak and mesodermal specification) at cysteine 466. Notably, polysulfide supplementation in iPSCs and subsequent induction of the mesoderm resulted in increased stability of the β-catenin protein, a key pathway that induces mesodermal lineage [23] (Fig. 1E). Although it increased WNT-specific mRNAs (Table 2), it reduced overall mesodermal specification identified by eomesodermin (EOMES) evaluation (Fig. 1F), possibly due to the dominant role of the OCT4 in stemness preservation. Taken together, these data suggest that increasing polysulfide availability prior to the specification events renders the cells resistant to mesodermal induction and preserves stem cell identity.

### 3.2 Pluripotent stem cell fate is sensitive to sulfide supplementation

**Fig. 2:**
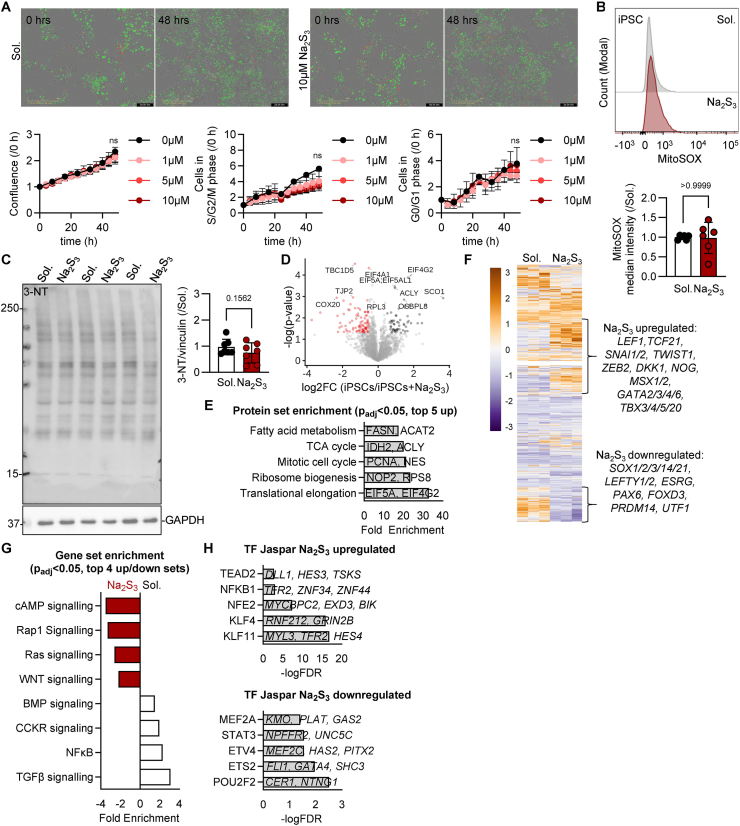
**Sulfide supplementation in iPSCs alters iPSCs persulfidome and transcriptional program.** **(A)** Normalized cell number in cell cycle phases detected by live cell imaging of FUCCI lentivirally induced iPSCs following treatment with 0–10 μmol/L Na_2_S_3_ for up to 48 h. **(B)** MitoSOX levels in iPSCs treated with solvent (Sol.) or with 5 μmol/L Na_2_S_3_ for 48 h. **(C)** Immunoblotting of 3-nitrotyrosine (3-NT) and vinculin (VIN) in iPSCs treated with solvent (Sol.) or with 5 μmol/L Na_2_S_3_ for 48 h. **(D)** Volcano plot showing persulfidated proteins in iPSCs treated with solvent (Sol.) or with 5 μmol/L Na_2_S_3_ for 48 h. **(E)** Enrichment analysis of proteins detected as in panel D. **(F)** Heatmap showing transcriptional changes following RNA sequencing in samples as in panel D. **(G)** Gene set enrichment analysis following RNA sequencing in samples as in panel D. **(H)** Transcription factor (TF) activities following RNA sequencing in samples as in panel D. 3 iPSC lines were used in 2–3 technical replicates. Graphs include the average of all experiments per line. Paired Student's t-test (B, C, D), Two-way ANOVA, Tukey's multiple comparison test (A).

**Fig. S1:**
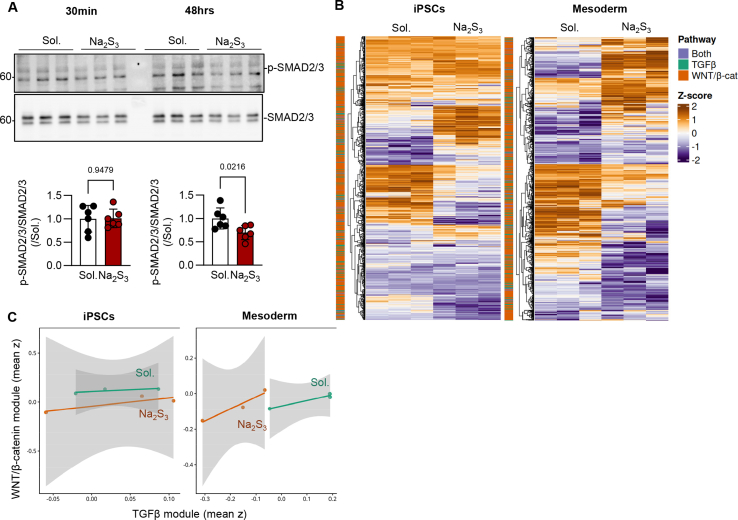
**Sulfide supplementation in TGFβ activation in pluripotency and mesodermalspecification.** (A) Immunoblotting of pSMAD2 (Ser465/467) /3 (Ser423/425), SMAD2/3 in iPSCs treated with solvent (Sol.) or with 5 μmol/L Na_2_S_3_ for 30 minutes or 48 hours. (B) Heatmap showing TGFβ and WNT signalling target mRNAs in iPSCs or mesodermal cells treated with solvent (Sol.) or with 5 μmol/L Na_2_S_3_ for 48 hours. (C) Scatter plots depict the relationship between TGFβ-associated and WNT/β-catenin-associated transcriptional module scores across individual samples (as in panel B) within each lineage under control (Sol, green) or Na₂S₃ treatment (orange) conditions. Each point represents one biological replicate. Linear regressions were fitted independently for each treatment, with shaded areas indicating the 95 % confidence interval of the slope. Annotated values inside each panel denote the Pearson correlation coefficient (r) and the corresponding p-value for the given lineage × treatment group. 3 iPSC lines were used in 2 technical replicates. Graphs include the average of all experiments per line. Paired Student’s t-test (A).

To understand in depth the impact of sulfide supplementation in pluripotent stem cell commitment programs, next we characterized the impact of sulfide supplementation in iPSCs. Notably, sulfide supplementation in different concentrations did not impact pluripotent stem cell self-renewal capacity and proliferation (Fig. 2A, Video S1A-S1B), neither significantly affected mitochondrial reactive oxygen species generation (Fig. 2B) or protein nitration (Fig. 2C), indicating that the sulfide effects are not attributing to modulation of the redox surveillance pathways in iPSCs. Persulfidation studies showed that sulfide supplementation has a minimal impact on the total persulfidation as only 57 proteins with at least one persulfidated cysteine where significantly enriched compared to the non-treated iPSCs (Fig. 2D–Table 1), with the majority of those involved in ribosomal biogenesis, translational elongation, mitotic cell cycle and metabolism (Fig. 2E). Again, persulfidation of stemness inducers as DPPA4 as well as key proliferation proteins, i.e. proliferating cell nuclear antigen (PCNA), were found in the polysulfide supplemented group, while no stemness inhibitors or proteins related to stem cell specification were identified. Given that DPPA4 maintains “bivalent” chromatin marks and protects these promoters from DNA methylation, keeping them accessible for future activation [24], we next evaluated whether such changes would potentially impact the iPSC transcription to enhance the activity of the key pluripotency transcriptional factors. Indeed, transcriptionally, sulfide supplementation resulted in gene alterations with transcripts mainly involved in WNT signaling mRNAs, a pathway that initiates specification and inhibits self-renewal and stemness [25] (Fig. 2F and G, Table 2). Notably, long term addition of the polysulfide donor resulted in a TGFβ transcriptional suppression (Fig. 2G) and inhibition which was also evident in the phosphorylation of the direct TGFβ targets SMAD2/3 [26] (Fig. S1A). SMAD2/3 while important for cellular reprogramming [27], they are implicated both in the maintenance and inhibition of pluripotency [28]. Transcriptional factor perturbations studies showed that sulfide supplementation enhanced the activity of OCT4, Krüppel-like factor 4 (KLF4) and TEA domain transcription factor 2 (TEAD2), while inhibiting transcriptional factors responsible for cardiac and vascular development as myocyte enhancer factor 2A (MEF2A), E26 transformation-specific transcription factor 2 (ETS2) and ETS variant transcription factor 4 (ETV4) (Fig. 2H). Taken together, these data support the role of sulfides in the transcriptional control of pluripotency.

Supplementary video related to this article can be found at doi:10.1016/j.redox.2025.103926

The following is/are the supplementary data related to this article:

Video s4 Video s4

Video s5 Video s5

### 3.3 Pluripotent stem cell fate commitment is sensitive to sulfide supplementation

**Fig. 3:**
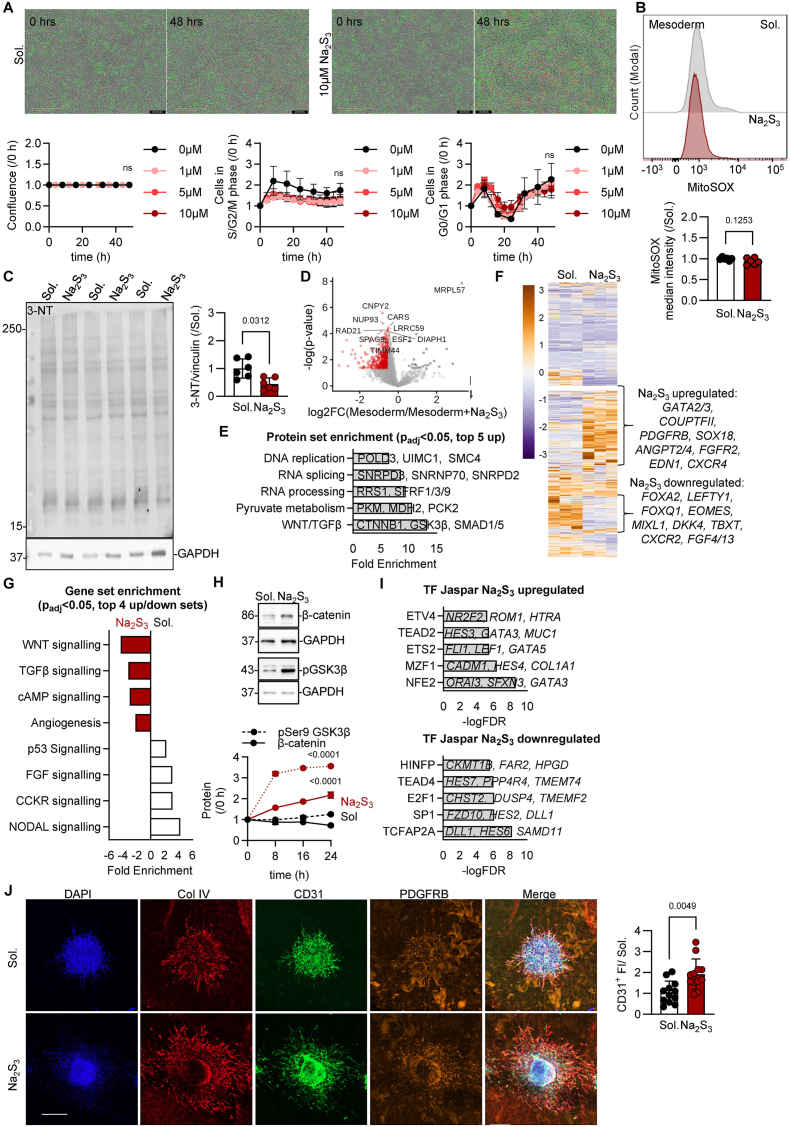
**Sulfide supplementation during mesodermal commitment promotes vascular development through activating WNT signaling, without impacting on redox balance.** **(A)** Normalized cell number in cell cycle phases detected by live cell imaging of FUCCI lentivirally induced mesodermal cells following treatment with 0–10 μmol/L Na_2_S_3_ for up to 48 h. **(B)** MitoSOX levels in mesodermal cells treated with solvent (Sol.) or with 5 μmol/L Na_2_S_3_ for 48 h. **(C)** Immunoblotting of 3-nitrotyrosine (3-NT) and vinculin (VIN) in mesodermal cells treated with solvent (Sol.) or with 5 μmol/L Na_2_S_3_ for 48 h. **(D)** Volcano plot showing persulfidated proteins in mesodermal cells treated with solvent (Sol.) or with 5 μmol/L Na_2_S_3_ for 48 h. **(E)** Enrichment analysis of proteins detected as in panel D. **(F)** Heatmap showing transcriptional changes following RNA sequencing in samples as in panel D. **(G)** Gene set enrichment analysis following RNA sequencing in samples as in panel D. **(H)** β-catenin stability and phosphorylation of GSK3β at Ser9 in mesodermal cells treated with solvent (Sol.) or with 5 μmol/L Na_2_S_3_ for 24 h and representative immunoblots at 24 h. **(I)** Transcription factor (TF) activities following RNA sequencing in samples as in panel D. **(J)** Representative immunofluorescent image of human iPSC-derived vascular organoids receiving solvent (Sol.) or 5 μmol/L Na_2_S_3_ after mesodermal specification. bar = 200μm. 3 iPSC lines were used in 2–3 technical replicates. Graphs include the average of all experiments per line. Paired Student's t-test (B, C, D), Two-way ANOVA, Tukey's multiple comparison test (A, H).

Last, we evaluated the impact of sulfide supplementation in the sulfide-deficient mesodermal lineage, hypothesizing that such an approach would potentially reverse specification and re-instate pluripotency. Similar to the pluripotent stem cells, sulfide supplementation did not alter proliferation and cell cycle of the mesodermal cells (Fig. 3A, Video S2A-S2B), neither affected mitochondrial reactive oxygen species levels (Fig. 3B), while the impact on protein nitration was minimal (Fig. 3C). While mesodermal cells showed reduced persulfidation of proteins, addition of sulfides enhanced persulfidation of 421 proteins in at least one cysteine (Fig. 3D–Table 1), which were involved in DNA replication/repair, the WNT/TGFβ pathway as well as cellular metabolism (Fig. 3E). Transcriptionally, sulfides reduced signaling transcripts of growth factors, i.e. fibroblast growth factor (FGF) and p53, while significantly increasing transcripts related to blood vessel morphogenesis and angiogenesis (Fig. 3F, Table 2), suggesting that once stem cells have specified to the mesodermal lineage, sulfide supplementation does not re-instate pluripotency, but induces further specification to the vasculature. In particular, mesodermal cells treated with sulfides for 48 h lost lineage specification markers and significantly gained endothelial cell transcription markers (Fig. 3G, Table 2). Such a result might be an effect of the persulfidation in the GSK3β and β-catenin pathways, as increased β-catenin stability initiated by de-phosphorylation through an inactive GSK3β, stimulates early WNT-driven endothelial commitment events and promotes endothelial gene expression [29]. Indeed, once sulfides were supplemented in mesodermal cells, GSK3β inhibitory phosphorylation at serine 9 was increased (Fig. 3H), resulting in increased β-catenin stability (Fig. 3H). Additionaly, the gain of TGFβ activation observed upon sulfide treatment may complement the WNT mediated endothelial specification (Fig. S1B, Table S1), consistent with evidence that SMAD2/3 promotes the expression of endothelial regulators such as ETV2 [[30], [31], [32]]. Interestingly, the correlation between TGFβ and WNT pathway enrichment module scores remained similar in iPSCs before and after sulfide exposure, mesodermal cells showed a stronger positive association between WNT and TGFβ pathways following sulfide supplementation (Fig. S1C), indicating a dominant WNT mediated endothelial specification cascade. Overall, TGFβ–WNT crosstalk is known to synergistically enhance vessel stabilization [33] yet their synergistic role in endothelial specification remains unkown. Hence, TGFβ activation in mesodermal cells likely contributes as a separate pathway to the pro-angiogenic and endothelial outcomes of sulfide supplementation. Transcription factor perturbation analysis pointed towards increased activity of the ETV4 and ETS2 transcriptional factors (Fig. 3I). Functionally, sulfide supplementation resulted in organoids exhibiting two times more CD31^+^ vessels compared to the respective solvent treated vascular organoids (Fig. 3J).

Supplementary video related to this article can be found at doi:10.1016/j.redox.2025.103926

The following is/are the supplementary data related to this article:

Video s6 Video s6

Video s7 Video s7

## 4 Conclusion

Our work identifies endogenous sulfide signaling via protein persulfidation as a switch that separates pluripotency from lineage commitment and that operates in a time-dependent manner. In iPSCs, the sulfide/persulfide axis reinforces stemness, whereas after entry into mesoderm it rewires toward pathways that bias endothelial output. Thus, sulfide availability does not simply scale redox tone, it programs fate by selectively modifying nodes in the pluripotency and WNT/BMP networks.

Antioxidant defenses are increasingly recognized as active regulators, not just bystanders of stem cell identity [34,35]. Pluripotent cells maintain a low, highly buffered redox setpoint through robust glutathione and thioredoxin/peroxiredoxin systems [36], restrained mitochondrial respiration and nuclear factor erythroid 2-related factor 2 (NRF2)-dependent programs, which favors OCT4/SOX2/NANOG activity and self-renewal [37]. In contrast, transient increases in reactive oxygen species (ROS) act as instructive cues that bias early lineage decisions (e.g. toward mesendoderm or hematopoietic fates), while sustained oxidative pressure accelerates exit from pluripotency [38,39]. Mechanistically, cysteine-based oxidative post-translational modifications (oxPTMs), including *S*-sulfenylation, *S*-nitrosylation, and *S*-glutathionylation, have been reported on transcription factors and chromatin regulators implicated in stemness and differentiation and on key metabolic and cytoskeletal proteins that shape fate transitions [40]. Persulfidation (*S*-sulfhydration) adds a distinct layer to this code: it can shield catalytic/structural thiols from over-oxidation, re-tune protein–protein and protein–DNA interactions and its age-related decline has been linked to impaired proteostasis, mitochondrial dysfunction and diminished tissue repair in terminally differentiated cells (e.g. vasculature, heart, brain) [5,[41], [42], [43]]. Here we report a dynamic regulation of protein persulfidation during early mesodermal specification. Although, mesodermal lineage shows reduced cystine derived sulfides and as such a global loss of protein persulfidation would have been expected - this was not the case. One possible explanation for the selective increase in persulfidation in a subset of proteins within the mesodermal lineage is that early mesodermal cells, as a highly plastic cell type, exhibit elevated autophagic flux compared to terminally differentiated cells [[44], [45], [46]], which could enhance spatial intracellular cysteine and sulfur availability to modify specific proteins. Alternatively, differential regulation of sulfur transferases/persulfidases i.e. 3 mercapropyruvate sulfur transferase [47] or persulfide-removing enzymes, which up to date remain unkown, during differentiation, may contribute to the stability and selective enrichment of persulfidated cysteines in these contexts. In contrast to the regulation of persulfidation in terminally differentiated cells and in disease settings which heavily relies on the expression of the key sulfide generating enzymes [2,14]; during differentiation, fluctuations in global persulfidation may not simply reflect their changes. Shifts in sulfide generating enzymatic activities, shifts in the redox/pH environment, metabolite levels (e.g. cysteine, glutathione, homocysteine), or mitochondrial sulfide clearance capacity (e.g. SQR activity) could all contribute to the observed persulfidation changes. Indeed, analogous dynamic regulation of redox balance and thiol modifications has been documented during stem cell differentiation, in which intracellular pH and redox potential shift with lineage commitment (e.g. iPSC pH/redox changes) [48]. Importantly, a direct, causal map from persulfidation to large-scale transcriptional reprogramming in pluripotent cells remains unestablished, most evidence is associative or limited to single targets. Against this backdrop, the WNT/β-catenin/GSK3β axis provides a well-defined biochemical lever through which redox signaling can influence fate: stabilizing β-catenin (e.g. via GSK3β inhibition) promotes primitive-streak/mesoderm entry in human primed iPSCs and guides vascular specification in a time-dependent manner, whereas β-catenin destabilization or premature pathway inhibition diverts or delays these outcomes. Our study situates persulfidation within this framework as an inhibitor of GSK3β, similarly to previous reports in the context of Alzheimer's Disease [49], suggesting that it is a tractable redox modification capable of interfacing with canonical fate-setting pathways while highlighting the unmet need to connect discrete oxPTMs to genome-wide transcriptional programs. Our findings align with the view that canonical WNT/β-catenin signaling and SMAD drive primitive streak/mesoderm formation, while GSK3β acts as a gatekeeper of β-catenin stability, transient WNT activation can sustain stemness in naïve contexts but pushes human primed iPSCs towards mesodermal lineage commitment. In this context, the transient activation of TGFβ signaling following sulfide supplementation may contribute to the coordinated modulation of WNT/β-catenin activity, helping to preserve a transcriptional landscape permissive for pluripotency while maintaining responsiveness to mesodermal cues. They also dovetail with literature placing OCT4/POU5F1 at the core of the pluripotency circuitry and DPPA4 as a chromatin-priming factor that preserves promoter competence. By showing that sulfides directly modulate these nodes (e.g. OCT4 and components of the GSK3β/β-catenin axis), our study adds a chemical biology layer to the transcriptional and signaling frameworks that govern early fate decisions.

Conceptually, these results position sulfide biology as a tunable handle for stem cell engineering: timed modulation of polysulfide availability could help preserve pluripotency when desired, or after mesoderm entry promote vascular endothelial specification and vessel formation. Practically, this suggests window-specific dosing strategies for organoid vascularization and regenerative applications. Future work should test causality with site-directed mutants of the identified cysteines (e.g. OCT4, GSK3β, β-catenin), resolve upstream metabolic sources of sulfide in each state and map whether analogous persulfidation logic extends to other germ-layer trajectories.

## Funding

This work was supported by the 10.13039/501100001659Deutsche Forschungsgemeinschaft (CRC1366/2 Project B01 to S.-I.B. and Project B07 to R.O; Project ID 39404578; the Emmy Noether Programme BI 2163/1–2 to S.-I.BCRC1531, Project A02 to S.-I.B. and Project S01 to I.W., Project ID: 456687919).; the 10.13039/501100009052Hector Fellow Academy to S.-I.B.; the National Natural Science Foundation of China: Project IDs; 82070501, 82271479 and 32350021 to J.H., and a scholarship from Onnasis Foundation to M.-K.D.

## CRediT authorship contribution statement

**Janina Wittig:** Conceptualization, Data curation, Formal analysis, Investigation, Methodology, Supervision, Visualization, Writing – original draft, Writing – review & editing. **Ran Xu:** Data curation, Formal analysis, Investigation, Methodology, Writing – review & editing. **Fredy Delgado Lagos:** Formal analysis, Methodology, Writing – review & editing. **Maria-Kyriaki Drekolia:** Data curation, Formal analysis, Methodology, Writing – review & editing. **Boran Zhang:** Data curation, Formal analysis, Methodology, Writing – review & editing. **Ioannis Theodorou:** Data curation, Formal analysis, Methodology, Writing – review & editing. **Yunyun Chen:** Methodology, Writing – review & editing. **Yali Du:** Methodology, Writing – review & editing. **Lavanya Gupta:** Methodology, Writing – review & editing. **Cui Hanyu:** Methodology, Writing – review & editing. **Li Yuanyuan:** Methodology, Writing – review & editing. **Cheng Bo:** Methodology, Writing – review & editing. **Stefan Günther:** Data curation, Formal analysis, Methodology, Writing – review & editing. **Ilka Wittig:** Data curation, Formal analysis, Methodology, Writing – review & editing. **Roxana Ola:** Funding acquisition, Investigation, Writing – review & editing. **Jiong Hu:** Funding acquisition, Supervision, Writing – review & editing. **Sofia-Iris Bibli:** Formal analysis, Resources, Supervision, Validation, Visualization, Writing – original draft, Writing – review & editing.

## Declaration of competing interest

There are no conflicts of interest to declare.

## Data Availability

The authors declare that the data supporting the findings of this study are available within the paper. Requests for material can be made to the corresponding authors. RNAseq data are deposited in ENA under the accession number PRJEB101608. Proteomics data are deposited in PRIDE under the accession number PXD071015.

## References

[1] Jin Y.-Q., Yuan H., Liu Y.-F., Zhu Y.-W., Wang Y., Liang X.-Y., Gao W., Ren Z.-G., Ji X.-Y., Wu D.-D. (2020). Role of hydrogen sulfide in health and disease. MedComm.

[2] Zivanovic J., Kouroussis E., Kohl J.B., Adhikari B., Bursac B., Schott-Roux S., Petrovic D., Miljkovic J.L., Thomas-Lopez D., Jung Y., Miler M., Mitchell S., Milosevic V., Gomes J.E., Benhar M., Gonzalez-Zorn B., Ivanovic-Burmazovic I., Torregrossa R., Mitchell J.R., Whiteman M., Schwarz G., Snyder S.H., Paul B.D., Carroll K.S., Filipovic M.R. (2020). Selective persulfide detection reveals evolutionarily conserved antiaging effects of S-Sulfhydration. Cell Metab..

[3] Drekolia M.-K., Karantanou C., Wittig I., Li Y., Fuhrmann D.C., Brüne B., Katsouda A., Hu J., Papapetropoulos A., Bibli S.-I. (2024). Loss of cardiac mitochondrial complex I persulfidation impairs NAD+ homeostasis in aging. Redox Biol..

[4] Luo S., Kong C., Ye D., Liu X., Wang Y., Meng G., Han Y., Xie L., Ji Y. (2023). Protein persulfidation: recent progress and future directions. Antioxidants Redox Signal..

[5] Vignane T., Filipovic M.R. (2023). Emerging chemical biology of protein persulfidation. Antioxidants Redox Signal..

[6] Liu Y., Yang R., Liu X., Zhou Y., Qu C., Kikuiri T., Wang S., Zandi E., Du J., Ambudkar I.S., Shi S. (2014). Hydrogen sulfide maintains mesenchymal stem cell function and bone homeostasis via regulation of Ca(2+) channel sulfhydration. Cell Stem Cell.

[7] Zhao Y., Wei H., Kong G., Shim W., Zhang G. (2013). Hydrogen sulfide augments the proliferation and survival of human induced pluripotent stem cell-derived mesenchymal stromal cells through inhibition of BKCa. Cytotherapy.

[8] Abdelmonem M., Shahin N.N., Rashed L.A., Amin H.A.A., Shamaa A.A., Shaheen A.A. (2019). Hydrogen sulfide enhances the effectiveness of mesenchymal stem cell therapy in rats with heart failure: in vitro preconditioning versus in vivo co-delivery. Biomed. Pharmacother..

[9] Liu D., Wang Z., Zhan J., Zhang Q., Wang J., Zhang Q., Xian X., Luan Q., Hao A. (2014). Hydrogen sulfide promotes proliferation and neuronal differentiation of neural stem cells and protects hypoxia-induced decrease in hippocampal neurogenesis. Pharmacol. Biochem. Behav..

[10] Pera M.F., Rossant J. (2021). The exploration of pluripotency space: charting cell state transitions in peri-implantation development. Cell Stem Cell.

[11] Wells J.M., Watt F.M. (2018). Diverse mechanisms for endogenous regeneration and repair in mammalian organs. Nature.

[12] Gu M. (2018). Efficient differentiation of human pluripotent stem cells to endothelial cells. Curr. Protoc. Hum. Genet..

[13] Barayeu U., Schilling D., Eid M., Da Xavier Silva T.N., Schlicker L., Mitreska N., Zapp C., Gräter F., Miller A.K., Kappl R., Schulze A., Friedmann Angeli J.P., Dick T.P. (2023). Hydropersulfides inhibit lipid peroxidation and ferroptosis by scavenging radicals. Nat. Chem. Biol..

[14] Bibli S.-I., Hu J., Looso M., Weigert A., Ratiu C., Wittig J., Drekolia M.K., Tombor L., Randriamboavonjy V., Leisegang M.S., Goymann P., Delgado Lagos F., Fisslthaler B., Zukunft S., Kyselova A., Justo A.F.O., Heidler J., Tsilimigras D., Brandes R.P., Dimmeler S., Papapetropoulos A., Knapp S., Offermanns S., Wittig I., Nishimura S.L., Sigala F., Fleming I. (2021). Mapping the endothelial cell S-sulfhydrome highlights the crucial role of integrin sulfhydration in vascular function. Circulation.

[15] Hu J., Leisegang M.S., Looso M., Drekolia M.-K., Wittig J., Mettner J., Karantanou C., Kyselova A., Dumbovic G., Li X., Li Y., Guenther S., John D., Siragusa M., Zukunft S., Oo J.A., Wittig I., Hille S., Weigert A., Knapp S., Brandes R.P., Müller O.J., Papapetropoulos A., Sigala F., Dobreva G., Kojonazarov B., Fleming I., Bibli S.-I. (2023). Disrupted binding of cystathionine γ-lyase to p53 promotes endothelial senescence. Circ. Res..

[16] Cao M., Li M., Li X., Li Y., Chen Y., Drekolia M.-K., Cheng X., Lagos F.D., Bibli S.-I., Hu J. (2025). Endothelial soluble epoxide hydrolase links polyunsaturated fatty acid metabolism to oxidative stress and atherosclerosis progression. Redox Biol..

[17] Hu J., Leisegang M.S., Looso M., Drekolia M.-K., Wittig J., Mettner J., Karantanou C., Kyselova A., Dumbovic G., Li X., Li Y., Guenther S., John D., Siragusa M., Zukunft S., Oo J.A., Wittig I., Hille S., Weigert A., Knapp S., Brandes R.P., Müller O.J., Papapetropoulos A., Sigala F., Dobreva G., Kojonazarov B., Fleming I., Bibli S.-I. (2023). Disrupted binding of cystathionine γ-lyase to p53 promotes endothelial senescence. Circ. Res..

[18] Wimmer R.A., Leopoldi A., Aichinger M., Kerjaschki D., Penninger J.M. (2019). Generation of blood vessel organoids from human pluripotent stem cells. Nat. Protoc..

[19] Shi G., Jin Y. (2010). Role of Oct4 in maintaining and regaining stem cell pluripotency. Stem Cell Res. Ther..

[20] Marsboom G., Zhang G.-F., Pohl-Avila N., Zhang Y., Yuan Y., Kang H., Hao B., Brunengraber H., Malik A.B., Rehman J. (2016). Glutamine metabolism regulates the pluripotency transcription factor OCT4. Cell Rep..

[21] Klein R.H., Tung P.-Y., Somanath P., Fehling H.J., Knoepfler P.S. (2018). Genomic functions of developmental pluripotency associated factor 4 (Dppa4) in pluripotent stem cells and cancer. Stem Cell Res..

[22] Tang B., Raviv A., Esposito D., Flanders K.C., Daniel C., Nghiem B.T., Garfield S., Lim L., Mannan P., Robles A.I., Smith W.I., Zimmerberg J., Ravin R., Wakefield L.M. (2015). A flexible reporter system for direct observation and isolation of cancer stem cells. Stem Cell Rep..

[23] Lindsley R.C., Gill J.G., Kyba M., Murphy T.L., Murphy K.M. (2006). Canonical Wnt signaling is required for development of embryonic stem cell-derived mesoderm. Development.

[24] Eckersley-Maslin M.A., Parry A., Blotenburg M., Krueger C., Ito Y., Franklin V.N.R., Narita M., D'Santos C.S., Reik W. (2020). Epigenetic priming by Dppa2 and 4 in pluripotency facilitates multi-lineage commitment. Nat. Struct. Mol. Biol..

[25] Sato N., Meijer L., Skaltsounis L., Greengard P., Brivanlou A.H. (2004). Maintenance of pluripotency in human and mouse embryonic stem cells through activation of Wnt signaling by a pharmacological GSK-3-specific inhibitor. Nat. Med..

[26] Derynck R., Zhang Y.E. (2003). Smad-dependent and Smad-independent pathways in TGF-beta family signalling. Nature.

[27] Ruetz T., Pfisterer U., Di Stefano B., Ashmore J., Beniazza M., Tian T.V., Kaemena D.F., Tosti L., Tan W., Manning J.R., Chantzoura E., Ottosson D.R., Collombet S., Johnsson A., Cohen E., Yusa K., Linnarsson S., Graf T., Parmar M., Kaji K. (2017). Constitutively active SMAD2/3 are broad-scope potentiators of transcription-factor-mediated cellular reprogramming. Cell Stem Cell.

[28] Watabe T., Miyazono K. (2009). Roles of TGF-beta family signaling in stem cell renewal and differentiation. Cell Res..

[29] Corada M., Nyqvist D., Orsenigo F., Caprini A., Giampietro C., Taketo M.M., Iruela-Arispe M.L., Adams R.H., Dejana E. (2010). The Wnt/beta-catenin pathway modulates vascular remodeling and specification by upregulating Dll4/Notch signaling. Dev. Cell.

[30] Zhao H., Choi K. (2017). A CRISPR screen identifies genes controlling Etv2 threshold expression in murine hemangiogenic fate commitment. Nat. Commun..

[31] Pardali E., Goumans M.-J., ten Dijke P. (2010). Signaling by members of the TGF-beta family in vascular morphogenesis and disease. Trends Cell Biol..

[32] Howell E.D., Yzaguirre A.D., Gao P., Lis R., He B., Lakadamyali M., Rafii S., Tan K., Speck N.A. (2021). Efficient hemogenic endothelial cell specification by RUNX1 is dependent on baseline chromatin accessibility of RUNX1-regulated TGFβ target genes. Genes Dev..

[33] DiRenzo D.M., Chaudhary M.A., Shi X., Franco S.R., Zent J., Wang K., Guo L.-W., Kent K.C. (2016). A crosstalk between TGF-β/Smad3 and Wnt/β-catenin pathways promotes vascular smooth muscle cell proliferation. Cell. Signal..

[34] Bigarella C.L., Liang R., Ghaffari S. (2014). Stem cells and the impact of ROS signaling. Development.

[35] Tan D.Q., Suda T. (2018). Reactive oxygen species and mitochondrial homeostasis as regulators of stem cell fate and function. Antioxidants Redox Signal..

[36] Jung H., Kim D.O., Byun J.-E., Kim W.S., Kim M.J., Song H.Y., Kim Y.K., Kang D.-K., Park Y.-J., Kim T.-D., Yoon S.R., Lee H.G., Choi E.-J., Min S.-H., Choi I. (2016). Thioredoxin-interacting protein regulates haematopoietic stem cell ageing and rejuvenation by inhibiting p38 kinase activity. Nat. Commun..

[37] Ji A.-R., Ku S.-Y., Cho M.S., Kim Y.Y., Kim Y.J., Oh S.K., Kim S.H., Moon S.Y., Choi Y.M. (2010). Reactive oxygen species enhance differentiation of human embryonic stem cells into mesendodermal lineage. Exp. Mol. Med..

[38] Ludin A., Gur-Cohen S., Golan K., Kaufmann K.B., Itkin T., Medaglia C., Lu X.-J., Ledergor G., Kollet O., Lapidot T. (2014). Reactive oxygen species regulate hematopoietic stem cell self-renewal, migration and development, as well as their bone marrow microenvironment, Antioxid. Redox Signal..

[39] Ulfig A., Jakob U. (2024). Redox heterogeneity in mouse embryonic stem cells individualizes cell fate decisions. Dev. Cell.

[40] Lee B.W.L., Ghode P., Ong D.S.T. (2019). Redox regulation of cell state and fate. Redox Biol..

[41] Bithi N., Link C., Henderson Y.O., Kim S., Yang J., Li L., Wang R., Willard B., Hine C. (2021). Dietary restriction transforms the mammalian protein persulfidome in a tissue-specific and cystathionine γ-lyase-dependent manner. Nat. Commun..

[42] He B., Zhang Z., Huang Z., Duan X., Wang Y., Cao J., Li L., He K., Nice E.C., He W., Gao W., Shen Z. (2023). Protein persulfidation: rewiring the hydrogen sulfide signaling in cell stress response. Biochem. Pharmacol..

[43] Yang C.-T., Devarie-Baez N.O., Hamsath A., Fu X.-D., Xian M. (2020). S-Persulfidation: Chemistry, chemical biology, and significance in health and disease. Antioxidants Redox Signal..

[44] Mizushima N., Levine B. (2010). Autophagy in mammalian development and differentiation. Nat. Cell Biol..

[45] Ho T.T., Warr M.R., Adelman E.R., Lansinger O.M., Flach J., Verovskaya E.V., Figueroa M.E., Passegué E. (2017). Autophagy maintains the metabolism and function of young and old stem cells. Nature.

[46] García-Prat L., Martínez-Vicente M., Perdiguero E., Ortet L., Rodríguez-Ubreva J., Rebollo E., Ruiz-Bonilla V., Gutarra S., Ballestar E., Serrano A.L., Sandri M., Muñoz-Cánoves P. (2016). Autophagy maintains stemness by preventing senescence. Nature.

[47] Pedre B., Talwar D., Barayeu U., Schilling D., Luzarowski M., Sokolowski M., Glatt S., Dick T.P. (2023). 3-Mercaptopyruvate sulfur transferase is a protein persulfidase. Nat. Chem. Biol..

[48] Giallongo S., Rehakova D., Raffaele M., Lo Re O., Koutna I., Vinciguerra M. (2021). Redox and epigenetics in human pluripotent stem cells differentiation. Antioxidants Redox Signal..

[49] Giovinazzo D., Bursac B., Sbodio J.I., Nalluru S., Vignane T., Snowman A.M., Albacarys L.M., Sedlak T.W., Torregrossa R., Whiteman M., Filipovic M.R., Snyder S.H., Paul B.D. (2021). Hydrogen sulfide is neuroprotective in Alzheimer's disease by sulfhydrating GSK3β and inhibiting Tau hyperphosphorylation. Proc. Natl. Acad. Sci. U. S. A.

